# The role of CD4^+^ T cells in tumor and chronic viral immune responses

**DOI:** 10.1002/mco2.390

**Published:** 2023-10-10

**Authors:** Luoyingzi Xie, Jingyi Fang, Juncheng Yu, Weinan Zhang, Zhiqiang He, Lilin Ye, Huaizhi Wang

**Affiliations:** ^1^ Institute of Hepatopancreatobiliary Surgery Chongqing General Hospital Chongqing China; ^2^ The Institute of Immunology Third Military Medical University (Army Medical University) Chongqing China; ^3^ Department of Thoracic Surgery Xinqiao Hospital Third Military Medical University (Army Medical University) Chongqing China; ^4^ Department of Plastic & Cosmetic Surgery Army Medical Center of PLA Amy Medical University Chongqing China

**Keywords:** CD4^+^ T cells, chronic viral infection, immunotherapy, tumor microenvironment

## Abstract

Immunotherapies are mainly aimed to promote a CD8^+^ T cell response rather than a CD4^+^ T cell response as cytotoxic T lymphocytes (CTLs) can directly kill target cells. Recently, CD4^+^ T cells have received more attention due to their diverse roles in tumors and chronic viral infections. In antitumor and antichronic viral responses, CD4^+^ T cells relay help signals through dendritic cells to indirectly regulate CD8^+^ T cell response, interact with B cells or macrophages to indirectly modulate humoral immunity or macrophage polarization, and inhibit tumor blood vessel formation. Additionally, CD4^+^ T cells can also exhibit direct cytotoxicity toward target cells. However, regulatory T cells exhibit immunosuppression and CD4^+^ T cells become exhausted, which promote tumor progression and chronic viral persistence. Finally, we also outline immunotherapies based on CD4^+^ T cells, including adoptive cell transfer, vaccines, and immune checkpoint blockade. Overall, this review summarizes diverse roles of CD4^+^ T cells in the antitumor or protumor and chronic viral responses, and also highlights the immunotherapies based on CD4^+^ T cells, giving a better understanding of their roles in tumors and chronic viral infections.

## Graphical abstract

In this review, we summarize CD4^+^ T cells play multiple roles in tumor and chronic viral responses, including antitumor and antichronic viral roles and protumor and prochronic viral roles. In antitumor and antichronic viral responses, CD4^+^ T cells provide indirect assistance to the cytotoxic CD8^+^ T cells relayed by DCs, interact with B cells, macrophages and NK cells to indirectly modulate response, and inhibit tumor blood vessel formation. Additionally, cytotoxic CD4^+^ T cells can induce direct cytotoxicity toward target cells. However, CD4^+^ T cells also can mediate protumor and prochronic viral responses, such as exhausted CD4^+^ T cells and Treg cells. Overall, the roles played by CD4^+^ T cells in tumors and chronic viral infection are complex. TCR, T cell receptor; cDC1, classical dendritic cell 1; MHC, major histocompatibility complex; IFN, interferon; TNF, tumor necrosis factor; TGF, transforming growth factor.

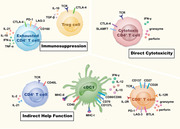

## 1 INTRODUCTION

Over the past few years, tumor immunotherapies, such as adoptive cell therapy and immune checkpoint blockade (ICB), had already become emerging strategies in the antitumor response and antichronic viral response. Most of the immunotherapies are based on CD8^+^ T cells rather than CD4^+^ T cells, as CD8^+^ T cells are considered to be more efficient at killing target cells. In addition, opposed to the major histocompatibility complex (MHC) class I molecules, MHC class II molecules are almost absent in most of tumor cells. However, due to continuous antigen stimulation in tumor microenvironment (TME) and chronic viral infections, most of CD8^+^ T cells become exhausted.^1^, ^2^ The process usually occurs in a hierarchical manner, starting with a significant reduction in interleukin (IL)‐2 (IL‐2) expression, which is essential for T‐cell survival and proliferation, thus making it difficult to maintain their activation state.^3^, ^4^ Furthermore, tumor necrosis factor (TNF), interferon‐γ (IFN‐γ), chemokines, and granzymes produced by activated T cells decrease sharply resulting in a loss of antitumor and antichronic viral function.^5^ Eventually, if the strong antigen stimulation persists, the specific cytotoxic T lymphocytes (CTLs) tend to disappear, especially in the absence of help from CD4^+^ T cells.^4^, ^6^, ^7^ Along with “functional exhaustion,” exhausted CTLs specifically coexpress high levels of inhibitory receptors, including programmed cell death protein 1 (PD‐1), cytotoxic T‐lymphocyte associated antigen‐4 (CTLA‐4), lymphocyte activation gene‐3 (LAG‐3), T cell immunoglobulin and mucin domain‐containing protein‐3 (TIM‐3), also known as immune checkpoints, which substantially affect the cytotoxic ability of CTLs and contribute to maintain their exhausted condition.^6^, ^8^, ^9^ In this case, recently, CD4^+^ T cells have received more attention.

Recently, CD4^+^ T cells have received increasing attention in tumors and chronic viral infections. According to previous studies, CD4^+^ T cells provide help through costimulatory molecules or cytokines to optimize the cytotoxicity and longevity of CD8^+^ T cells and regulate TME.^10^ Moreover, CD4^+^ T cells can also modulate other subsets to indirectly improve antitumor and antichronic viral responses, such as the interaction of CD4^+^ T cells with B cells,^11^ the regulation of macrophage polarization,^12^ and the modulation of vessel system formation in tumor tissue.^13^ In addition, cytotoxic CD4^+^ T cells have been proven to directly induce tumor cell apoptosis, mediated by granzymes and perforin.^14^ However, they also help form an immunosuppressive environment, which can prevent a favorable outcome in antitumor immunity.^15^

In this review, we mainly focus on how CD4^+^ T cells provide help to CD8^+^ T cells through dendritic cells (DCs) and other subsets in antitumor and antichronic viral immunity, how the cytotoxic CD4^+^ T cells directly kill target cells, and the immunosuppressive microenvironment mediated by CD4^+^ T cells in protumor and prochronic viral immunity. Meanwhile, we have also summarized antitumor and antichronic viral immunotherapies based on CD4^+^ T cells.

## 2 A BRIEF DESCRIPTION OF CD4^+^ T CELL CLASSIFICATION

**FIGURE 1:**
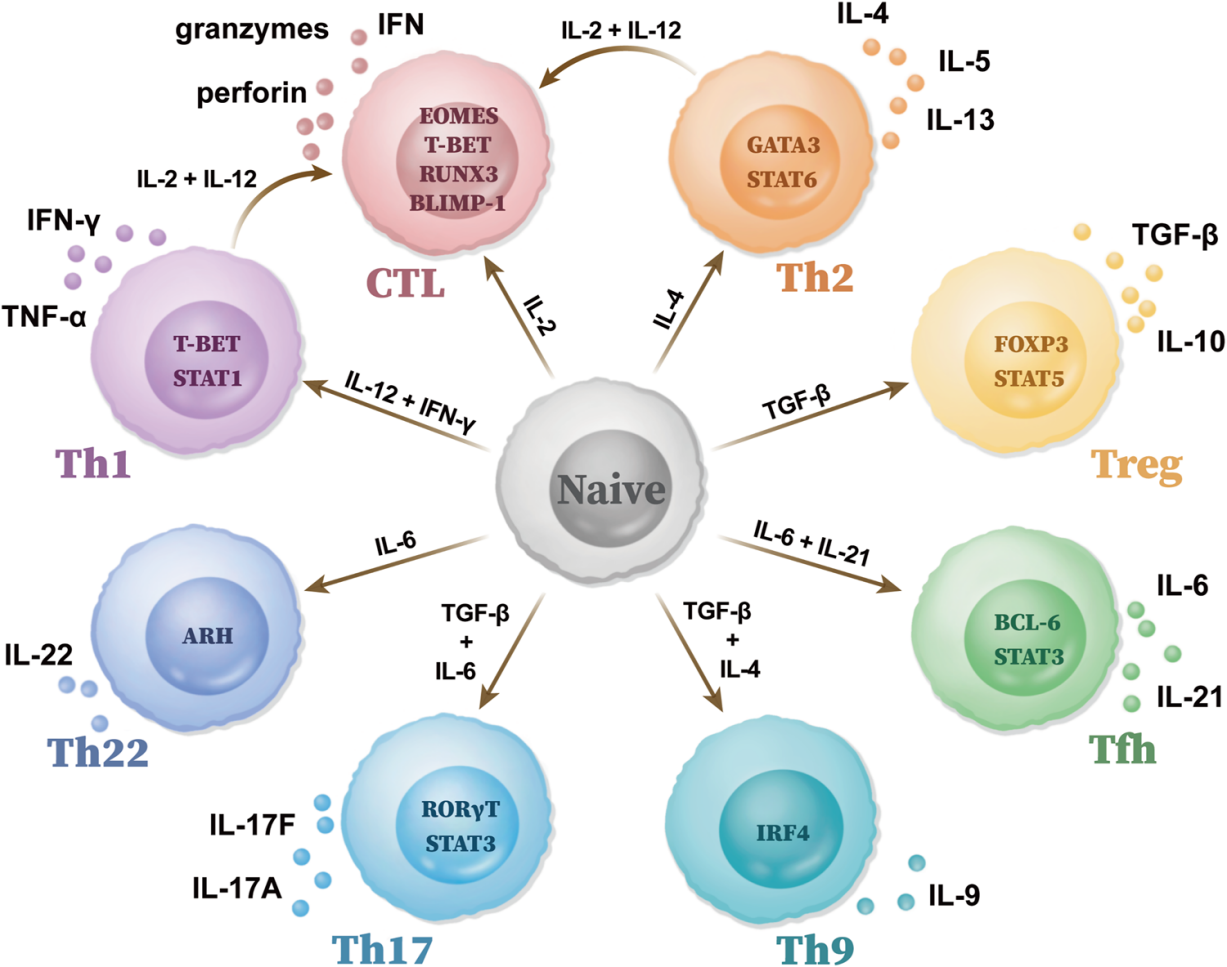
Classification and ontogeny of CD4^+^ T cells. Cytokines and transcription factors promote differentiation of naïve CD4^+^ T cells into several sub‐populations. According to the differences in expressions of transcriptional factors and functions, they are classified into Th1, Th2, Th9, Th17, Th22, Tfh, regulatory T cell (Treg), and cytotoxic CD4^+^ T cells. Specifically, the phenotype and killing function of cytotoxic CD4^+^ T cells in tumor microenvironment have been distinguished and represented by the expression of granzymes and/or perforin. CTL, cytotoxic T lymphocyte; Th1, T helper 1 cell; Th2, T helper 2 cell; Th9, T helper 9 cell; Th17, T helper 17 cell; Th22, T helper 22 cell; Tfh, follicular helper T cell; Treg, regulatory T cell; IFN, interferon; TNF, tumor necrosis factor; TGF‐β, transforming growth factor β.

Similar to CD8^+^ T cells, the recognition between TCR and specific epitopes binding in MHC‐II on the surface of antigen‐presenting cells (APCs) is first required for the priming of CD4^+^ T cells. Whereafter, multiple transcriptional programs mediated by different cytokines initiate the differentiation from naïve T cells to other sub‐populations in order to eliminate different pathogens. Depending on different phenotypes and functions, CD4^+^ T cells are mainly classified into helper T (Th) lymphocytes and regulatory T (Treg) lymphocytes. The Th cells provide help to enhance the immune effects of CD8^+^ T cells and B cells, while Treg cells exhibit an immunosuppressive effect. Th lymphocytes mainly include Th1, Th2, Th17, and Tfh, according to the specific expression of transcription factors and the different secreted cytokines (Figure 1).

### 2.1 Th1 cells

Naïve CD4^+^ T cells differentiate into Th1 cells with the expression of key transcription factors T‐box transcription factor 21 (T‐bet) and signal transducer and activator of transcription (STAT) family proteins, which are mediated by IL‐12 and IFN‐γ in response to intracellular pathogenic infections, inflammatory, and autoimmune diseases, such as asthma, inflammatory intestinal disorders, and lupus.^16^, ^17^, ^18^, ^19^ Especially, IL‐12 has been shown to have the ability to switch the Th17 cells to Th1 cells.^20^ Additionally, the differentiated Th2 cells transferred to lymphocytic choriomeningitis virus (LCMV)‐infected mice could acquire Th1 cell phenotypes, characterized by an increase in INF‐γ and a decrease in IL‐4.^21^ In addition to these main transcription factors, there are other transcriptional factors critical in the polarization of Th1 cells, such as interferon regulatory factor (IRF)1 at the downstream of INF‐γ^22^ and RUNX3, which could promote the expression of INF‐γ and suppress the expression of IL‐4.^23^, ^24^ IFN‐γ produced by Th1 cells, referred to as an important antitumor cytokine, can enhance the antitumor response through various ways, such as promoting the activation of CTLs, upregulating the expression of MHC molecules on DCs to improve the efficiency of antigen presentation, promoting the generation of M1‐like macrophages, inhibiting the suppressive microenvironment, and mediating the formation of the early vascular system in tumor tissue.^25^, ^26^ In addition, IFN‐γ is associated with a positive prognosis in ICB therapies^27^, ^28^, ^29^ and patients with nonresponsive melanoma exhibit a loss of IFN‐γ signaling.^30^ Similarly, a group of efficiently responsive CD4^+^ICOS^+^ (inducible co‐stimulator) T cells is identified as a phenotype of Th1 cells in bladder cancer patients who are all treated with Ipilimumab (anti‐CTLA‐4). This efficiently responsive sub‐population upregulates IFN‐γ expression under the help from ICOS pathway,^31^ which is also demonstrated in patients with metastatic melanoma^32^ and murine tumor models.^33^ Nevertheless, other studies have found that IFN‐γ in TME may also promote the expression levels of inhibitory receptors, such as PD‐1, PD‐L1, LAG‐3, and TIM‐3, which have been proven to help tumor escape.^27^, ^34^ While during antiviral infection, IFN‐γ can induce IFN‐stimulated genes (ISGs)^35^, ^36^ and promote IgG2a class switching, which subsequently exert antiviral effect.^37^ Additionally, the CD4^+^ T cell pools of HIV controllers are enriched in Th1 phenotype^38^, ^39^ and it is demonstrated that the decreased expression of ISG reduces the HIV sensitivity of Th1 cells.^40^

### 2.2 Th2 cells

Th2 cells are generated from Th0 cells in the presence of IL‐4 and characteristically express STAT6, which subsequently promotes the expression of GATA3 and inhibits the expression of Th1‐related transcriptional factors STAT4 and T‐bet. Additionally, Th2 cells have the capacity to secrete IL‐4, IL‐5, and IL‐13 to optimize humoral immunity and contribute to antipathogenic infections.^41^, ^42^, ^43^, ^44^ Previous research also demonstrated that Th2 cells could secrete IL‐4 and IL‐5 contributing to antitumor response by regulating TME and recruiting other effector cells in the tumor model with vaccination.^45^ Accordingly, in an IFN‐γ‐deficient murine model, the antitumor response significantly reduce in the absence of Th2 cytokines. Furthermore, tumor‐infiltrating eosinophils decrease in IL‐5‐deficient mice leading to a loss of antitumor response.^45^ Consistently, blocking IL‐5 pathway also contributes to tumor growth, while overexpressing IL‐5 or IL‐4 helps to predominantly improve eosinophils, macrophages infiltration to enhance tumor clearance.^45^, ^46^ In chimeric antigen receptor (CAR)‐T therapy, it was found that efficiently responsive cells not only had Th1‐associated phenotypes, but also could produce Th2‐related cytokines such as IL‐5 and IL‐13.^47^ However, Th2 cells have been reported to exhibit a protumor effect in breast and cervical tumors.^48^, ^49^ Moreover, type II immunity was found to promote tumor metastasis in colorectal, lung, and breast cancers.^50^, ^51^, ^52^, ^53^ In antiviral response, in addition to the positive effect induced by IL‐4, Th2 cells also play negative roles in multiple viral infections, including influenza virus,^54^, ^55^ vaccinia virus,^56^ respiratory syncytial virus (RSV),^57^ and herpes simplex virus (HSV) infections.^58^

### 2.3 Th9 cells

Th9 cells, which was initially considered as one of subpopulations in Th2 cells, have been distinguished due to the specific expression of IL‐9 recently. The transforming growth factor (TGF)‐β, IL‐4, and the transcription factor IRF4 are required for the Th9 cells, which can be involved in response to parasitic infections and allergic reaction.^59^ And in antitumor response, IL‐9 promotes the representing ability of DCs^60^ and mediates the mast cells to prevent tumor growth.^61^, ^62^ Moreover, adoptive transfer Th9 cells exhibit a robust antitumor immunity against melanoma.^63^ In addition, glucocorticoid‐induced tumor necrosis factor receptor family‐related protein (GITR) agonistic antibody DTA‐1 can induce IL‐9 to enhance the CTLs response by strengthening the function of DCs.^60^

### 2.4 Th17 cells

The polarization of Th17 cells, with the expression of transcription factors RORγt and STAT3 and the production of IL‐17A and IL‐17F, is induced by the combination of multiple cytokines.^64^, ^65^, ^66^, ^67^ In addition to the traditional related cytokines‐TGF‐β and IL‐6, the combination of IL‐23, IL‐6, and IL‐1β can also induce the production of IL‐17 even in the absence of TGF‐β.^65^ Th17 cells are considered to play a bifacial role in the response to extracellular infections and autoimmune diseases.^64^, ^65^, ^67^ In influenza and vaccinia viral infections, Th17 cells might contribute to immunopathology,^68^, ^69^ while they also exert protective functions by increasing CXC‐chemokines induced by IL‐17 to recruit neutrophils.^70^ Similarly, Th17 cells are also considered to play a bifacial role in antitumor immunity. The chronic proinflammatory effect of Th17 cells is closely related to the occurrence of some cancers.^71^, ^72^, ^73^, ^74^ Furthermore, a previous study demonstrated that IL‐17 could promote tumor progression by inducing the vascular system formation in tumor tissue.^75^ Additionally, Th17 cells have a positive role in antitumor immunity, which is mainly reflected in promoting the cell migration, such as NK cells and neutrophils, and regulating cell composition in the TME.^76^ Furthermore, Th17 cells can produce IL‐21, which contributes to the generation and infiltration of CX3CR1^+^CD8^+^ T cells to promote tumor clearance in the melanoma model.^77^

### 2.5 Th22

Different from other Th cells, Th22 cells were distinguished by the secreting of IL‐22, a member of IL‐10 family, and without the production of IL‐17, IL‐4, and IFN‐γ.^78^, ^79^ In human lymphocytes, during the generation of Th22 cells, which were thought to be connected with skin immunity,^78^, ^80^ the transcriptional factor aryl hydrocarbon receptor has been proved to play a significant role in upregulating the production of IL‐22 instead of IL‐17.^78^, ^81^ It has been demonstrated that Th22 cells and IL‐22 were associated with the progress of various human cancers such as gastric cancer,^82^ hepatocellular carcinoma,^83^ colon cancer,^84^ and lung cancer.^85^ During HIV infection, IL‐22‐produced cells appeared to sharply decrease, while the increase of IL‐22 could be observed after long‐term antiviral therapy.^86^ While in liver of mice and human with chronic hepatitis B viral infection, the expression of IL‐22 was corelated with the grade of inflammation and proliferation of liver stem/progenitor cells.^87^ Despite of the secreting of IL‐22, Th22 cells also express chemokine receptor C‐C chemokine receptor (CCR)6, CCR4, and CCR10.^79^

### 2.6 Tfh cells

Tfh cells with the production of IL‐4 and IL‐21 and the expression of Bcl‐6^88^, ^89^, ^90^ and STAT3 mediated by IL‐6 and IL‐21 are supposed to help the B cell maturity and germinal center (GC) formation.^11^, ^91^, ^92^ It has been proved that Tfh cells are the main source of IL‐21,^93^, ^94^ which is essential for the activation of B cells and the differentiation of plasma cells to assist humoral responses.^95^, ^96^, ^97^ In addition, Tfh cells can express ICOS and CD40L, which contribute to the interaction with B cells and play a significant role in the antitumor immune response.^94^, ^98^ The interaction of ICOS and ICOSL could activate the downstream P13K signaling, which is required for the differentiation of Tfh cells.^99^ Unlike other CD4^+^ T cells, Tfh cells can be distinguished by the expression of CXCR5,^100^ which is indirectly maintained by the expression of Bcl‐6.^101^ In murine tumor models, it was found that Tfh cells were closely linked with the recruitment of B cells and the formation of tertiary lymphoid structures (TLS), which subsequently affected the tumor control.^102^, ^103^ In breast cancer patients, the expression levels of Tfh gene signatures were correlated with TLS activity and clinical outcomes.^104^ Although the previous studies on CD4^+^ T cells in chronic infections have concentrated on Th1 cells, it has been proven that Tfh cells also play an important role in antiviral infection.^105^ For example, in LCMV and vaccinia virus infection models, the absence of Tfh cells led to a decrease in both quantity and quality of antiviral antibodies.^106^, ^107^ Likewise, the frequency of Tfh cells trends to increase after influenza vaccines injections in humans.^108^, ^109^

### 2.7 Treg cells

High concentration of TGF‐β is supposed to induce the expression of forkhead box P3 (FOXP3) and the polarization of Treg cells, which in turn secrete TGF‐β and IL‐10, exhibiting immune tolerance and immunosuppression.^15^, ^110^, ^111^, ^112^, ^113^ The expression of FOXP3 inhibits the expressions of IFN‐γ and IL‐2 and increases the expressions of CD25 and CTLA‐4, which help to maintain immunosuppression.^114^, ^115^, ^116^, ^117^ In addition to FOXP3, the expression of CD25 is also a biomarker in Treg cells. CD25, referred to as IL‐2Rα, can together with the β chain and γ chain of IL‐2 receptor, which competitively bind with IL‐2 to prevent further activation of effector T cells in the priming phase.^118^, ^119^, ^120^ Additionally, Treg cells also express the costimulatory receptor CTLA‐4, which competes with CD28 to bind to CD80/CD86 expressed on the surface of APCs, thereby reducing the CD28‐CD80/CD86 costimulation signal, which is required for the second step of T cell activation.^121^, ^122^, ^123^ Moreover, the expression of suppressor of cytokine signaling 1 (SOCS1) in Treg cells can inhibit the production of IFN‐γ and IL‐17 to sustain Foxp3 expression mediated by inhibiting the STAT1 and STAT3 signal pathways.^124^ TGF‐β and IL‐10 secreted by Treg cells also contribute to mediating an immunosuppressive TME.^125^, ^126^ It has already been reported that Treg cells are able to produce cytotoxic substances such as granzymes and perforin to kill antitumor effector cells, weakening the immune responses.^126^, ^127^, ^128^ Most studies on the impact of Treg cells in viral infections are based on chronic infections and the knowledge about their role in acute infections is not been well understood. It has been observed that the frequency of Treg cells increases in various human and animal viral infections. Accordingly, Treg cells can contribute to limiting the tissue immunoinflammatory damage, such as HSV‐induced eye damage, and also induce immune dysfunction in HCV and HIV infections.^129^

## 3 CD4^+^ T CELL ROLES IN ANTITUMOR AND ANTICHRONIC VIRAL RESPONSES

CD4^+^ T cells engage in antitumor and antichronic viral immune responses via several approaches, such as the indirect approaches and the direct approach. In indirect approaches, CD4^+^ T cells indirectly regulate CD8^+^ T cell response by DCs, interact with B cells or macrophages to indirectly modulate humoral immunity or macrophage polarization, and inhibit tumor blood vessel formation to promote antitumor response. Additionally, cytotoxic CD4^+^ T cells also can directly kill target cells to enhance antitumor immunity.

### 3.1 CD4^+^ T cell help shape CD8^+^ T cell response via DCs

CD8^+^ T cells activate and differentiate into cytotoxic CD8^+^ T cells after receiving MHC‐I signaling on tumor cells or APCs and in this process, DCs play a crucial role in priming CD8^+^ T cells due to their strong antigen presentation abilities.^130^, ^131^ DC subsets are categorized as plasmacytoid DCs, monocyte‐derived DCs, and classical DCs (cDCs) based on their developmental process, phenotypes, and function. cDCs can be further divided into two major subsets including CD8α^+^ and/or CD103^+^ cDC1s and CD11b^+^ cDC2s,^132^, ^133^ and both of them contribute to the immune response against tumor cells. cDC2s prefer to activate CD4^+^ T cells,^134^, ^135^, ^136^ whereas cDC1s mediate the priming of CD8^+^ T cells.^132^, ^133^, ^137^

**FIGURE 2:**
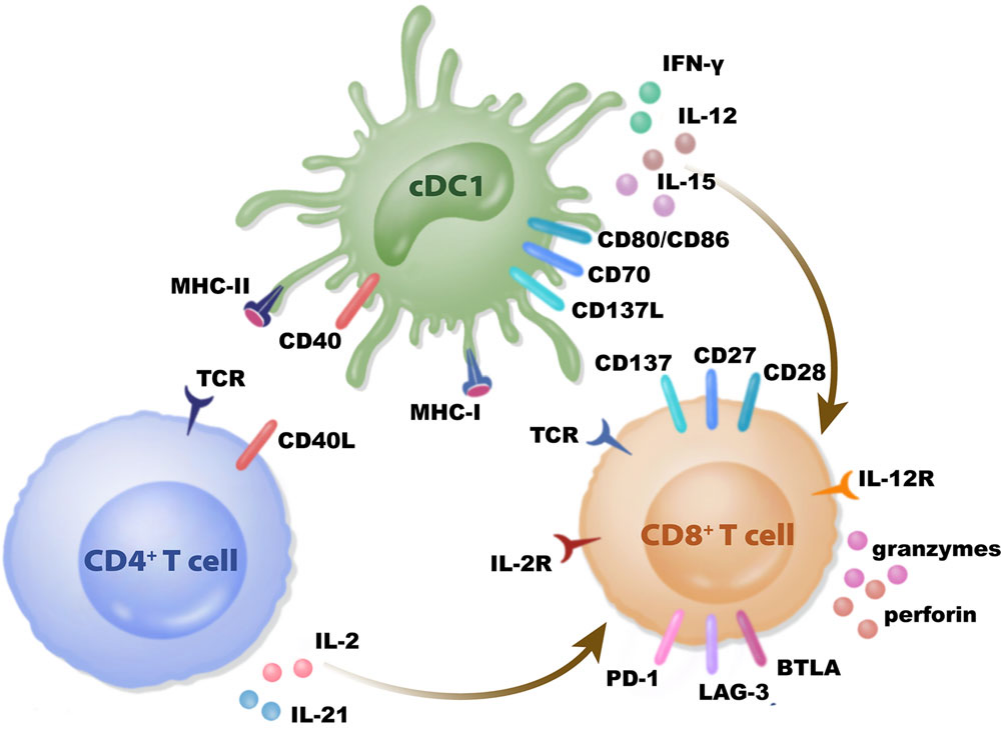
CD4^+^ T cells help CD8^+^ T cells to promote immune response by DCs. CD4^+^ T cells interact with cDC1s by costimulatory receptor CD40L/CD40 to regulate the priming and functional state of CD8^+^ T cells. In addition to CD40, the expression of costimulatory receptors on cDC1s, including CD70, CD80/CD86, and CD137L, plays a critical role in CTL activation. Moreover, the cytokines secreted from CD4^+^ T cells or DCs modulate CD8^+^ T cells response. DC, dendritic cell; cDC1, classical dendritic cell 1; TCR, T cell receptor; MHC, major histocompatibility complex.

Previous studies have proposed a two‐step model to understand the priming process of CD8^+^ T cells. The first step is referred as the priming of CD4^+^ T cells and CD8^+^ T cells through MHC‐II located on cDC2s and MHC‐I located on cDC1s, respectively.^138^, ^139^, ^140^ In the next step, activated CD8^+^ T cells secrete XC‐chemokine ligand 1 (XCL1), recruiting XC‐chemokine receptor 1 XCR1^+^ cDC1s, which sequentially interact with CD4^+^ T cells and CD8^+^ T cells and transmit the help signals to CD8^+^ T cells,^139^, ^140^ while the assistance from CD4^+^ T cells is highlighted in the second step in which the same cDC1 is required to present the same epitopes to both CD4^+^ T and CD8^+^ T cells. Considering the fact that specific antigens derived from endocytosed proteins are presented by MHC‐II on DCs after processing and also effectively presented by MHC‐I as a consequence of a particular phenomenon called antigen cross‐presentation in DCs^141^ — originally found in Bevan's research and named “cross‐priming” in 1976.^142^ During this time, DCs can relay help from CD4^+^ T cells to CD8^+^ T cells (Figure 2). Therefore, CD4^+^ T cell help is essential to prime CD8^+^ T cells by antigen cross‐presentation, even in MHC‐II‐negative tumor cells.^143^, ^144^, ^145^, ^146^ Parallel results were detected in vaccine models, suggesting the necessity of CD4^+^ T cell help while using long peptides that needed to be processed.^147^, ^148^

#### 3.1.1 DCs relay CD4^+^ T cell help through costimulatory molecules

The delivery of CD4^+^ T cell help in antigen cross‐presentation mainly relies on the interaction between CD40L on activated CD4^+^ T cells and CD40 on DCs.^143^ Studies on therapeutic antitumor vaccines have provided favorable evidence that, in the absence of CD40 signal, vaccination with MHC‐I‐binding peptides fails to induce an effective CTL response, while the vaccination with MHC‐I and MHC‐II‐binding peptides can overcome this situation.^147^, ^148^, ^149^ Besides, the expression of costimulatory receptors, including CD70, CD80/CD86, and CD137L, also plays an important role in CTL activation.^150^

The activation effects derived from the CD70 located on DCs interacting with the CD27 on CTLs in murine models are critical to delivering help to CD8^+^ T cells.^151^ The CD70–CD27 costimulation signal is well known to promote proliferation and differentiation toward effector or memory phenotypes in primed CD8^+^ T cells.^152^, ^153^ Many experiments also confirmed the requirement of the CD70–CD27 signal in CTL priming by using soluble CD70.^154^, ^155^ Moreover, blocking the CD70 signal effectively inhibits CD40‐mediated CTL priming,^156^ highlighting the critical role of the CD70 signal. CD27 costimulatory signals enhance T cell survival through antiapoptosis and prometabolism effects,^157^, ^158^ Additionally, CD27 costimulatory signals also increase the expression of IL‐12Rβ2, IL‐2Rα, and IL‐2 in activated CD8^+^ T cells, which drives the differentiation of CTLs.^151^, ^159^, ^160^

Besides the CD70–CD27 costimulatory signal, CD80/CD86–CD28 costimulation is an additional signal in CTL priming.^161^ CD28 induces the enhancement of the T cell receptor (TCR) signal, which subsequently primes the CD8^+^ T cell cycle and changes its metabolism to enhance the expansion of CD8^+^ T cells.^162^, ^163^ In antitumor vaccination, the combination of CD27 agonism and PD‐1 blockade effectively recapitulates CD4^+^ T cell help.^164^ Similarly, PD‐1 blockade promotes CD28 costimulatory signals,^165^ suggesting that CD27 and CD28 signals play a synergistic role in CD4^+^ T cell help.

#### 3.1.2 Cytokines from CD4^+^ T cells or DCs shape the CTL response

Besides the upregulation of costimulatory receptors, activated DCs with CD4^+^ T cell help can also increase the production of cytokines such as type I IFN, IL‐12, and IL‐15. It has already been proven that type I IFN and IL‐12 from activated DCs regulate TNF receptors and certain molecules related to CTL survival and functions.^166^ In the absence of type I IFN or IL‐12, the effector and memory functions of CD8^+^ T cells are compromised. Uniquely, IL‐15 mainly induces the differentiation of CTLs and promotes permanent survival based on inhibiting apoptosis mediated by TNF‐related apoptosis‐inducing ligand (TRAIL).^167^, ^168^, ^169^

CD4^+^ T cells can also deliver help to CD8^+^ T cells by IL‐2 and IL‐21 productions. CD4^+^ T cells‐derived IL‐2 is one of the most important cytokines as relevant research suggested that it was essential for promoting CD8^+^ T cells differentiation toward effector cytolytic T cells.^160^ In addition, IL‐2 signal mediates development of short‐lived effector CD8^+^ T cell response and is required for secondary expansion of CD8^+^ memory T cell.^170^, ^171^, ^172^ Besides IL‐2, CD4^+^ T cells can also provide IL‐21.^94^, ^173^, ^174^ In the absence of IL‐21, CTL response were impaired even with enhancement of CD4^+^ T cell help signals during chronic viral infections.^175^ Consistently, tumor‐infiltrating CD8^+^ T cells in an IL‐21R knockout mouse model exhibited inhibited granzyme B (GZMB) production.^94^

#### 3.1.3 The preponderances of helped CD8^+^ T cells

Activated CD8^+^ T cells differentiate into several subsets, including short‐lived effector T cells and long‐lived memory T cells.^176^, ^177^ In antitumor vaccination and virus infection models, the transcriptional differences in CTLs with the help of CD4^+^ T cells has been identified through RNA‐sequencing.^151^ Accordingly, compared with unhelped CD8^+^ T cells in mice only treated MHC‐I‐restricted epitope vaccination, the helped CD8^+^ T cells in mouse that received antitumor vaccine encoded MHC‐II and MHC‐I epitopes showed many discrepancies in master proteins about cytotoxicity and cell cycle—the upregulation of T‐BET and IRF4, and the downregulation of TCF‐7, EOMES, and ID3 were observed.^151^ Notably, T‐BET and EOMES expression levels determine the functional state of CTLs—T‐BET tends to drive CD8^+^ T cells to terminal differentiation‐effector phenotypes,^178^ while EOMES expression is upregulated during the effector to memory cell transition^179^—and ID3 is required for the generation of memory CD8^+^ T cells,^180^, ^181^ supporting the important role of CD4^+^ T cell help in formation of polyfunctional CTLs.

In addition, helped CTLs express higher effector molecules, including IFN‐γ, TNF, FASL, GZMA, and GZMB.^151^ Moreover, CTLs without the help from CD4^+^ T cells upregulate coinhibitory receptors, such as PD‐1, LAG‐3, and B and T lymphocyte attenuator (BTLA).^151^ Subsequently, blocking these three coinhibitory receptors in unhelped CD8^+^ T cells improves tumor control.^151^ Similar observation was found in a vaccination study that helpless CD8^+^ T cells exhibited reduced effector function and expressed higher levels of inhibitory receptors.^182^ These gene signatures are similar to those of exhausted T cells during chronic viral infections or tumors.^183^ Therefore, CD4^+^ T cell help could prevent CD8^+^ T cells from the exhausted phenotype.

The sequence analysis also demonstrated that helped CD8^+^ T cells gained a stronger extravasation potential by upregulation of chemokine receptors CXCR4 and CX3CR1,^151^ which could bind to CXCL12 and CX3CL1 from tumor cells, thus inducing CTLs infiltration into TME to exert antitumor effects.^184^, ^185^ The decreased density of infiltrating CD8^+^ T cells by CXCR4 and CX3CR1 inhibitors also supported this result.^151^ Except for chemokines, an increased expression of matrix metalloproteinases and a decreased expression of its inhibitory protein TIMIP2 were also found in helped CD8^+^ T cells,^151^ highlighting the important role of CD4^+^ T cells in promoting the penetration of CD8^+^ T cells into effector tissues.

As for memory CTLs, activated CD8^+^ T cells without help upregulated the expression of TRAIL, which mediated apoptosis during the secondary expansion of memory CTLs to restrict their proliferation.^186^, ^187^ In addition, helped CTLs expressed higher level of inhibitory apoptosis regulator BCL‐XL to amplify their survival and proliferation.^168^

### 3.2 Interaction of CD4^+^ T cells with macrophages, NK cells and B cells promotes the antitumor response

**FIGURE 3:**
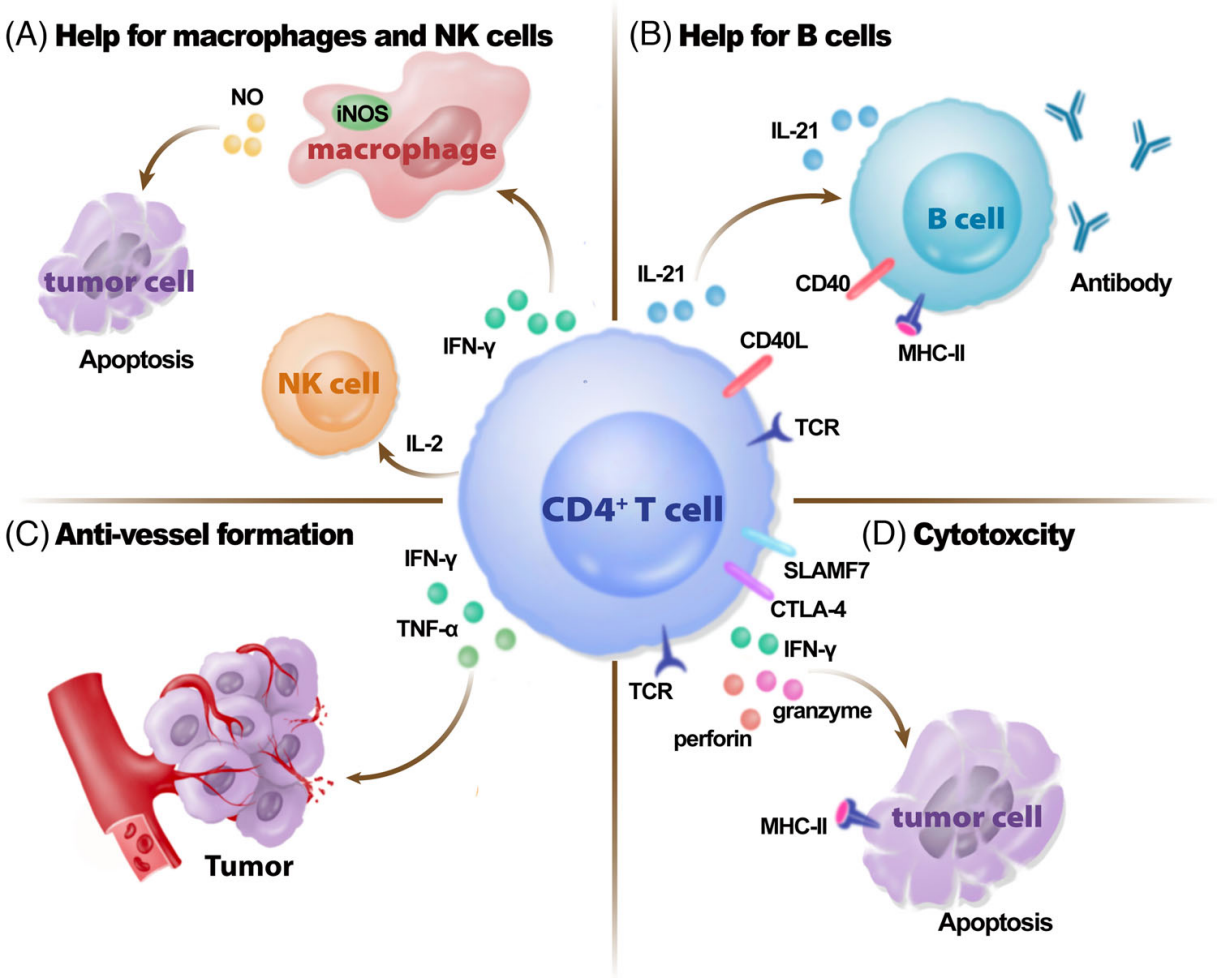
The indirect and direct functions of CD4^+^ T cells in antitumor and antichronic viral responses. (A–C) The indirect functions of CD4^+^ T cells in antitumor and antichronic viral response. (D) The direct cytotoxic function of CD4^+^ T cells. (A) CD4^+^ T cells can produce IFN‐γ and then upregulate the expression of iNOS in macrophages. iNOS in macrophages promotes the production of NO in TME and subsequently induces tumor cell apoptosis. CD4^+^ T cells can also help NK cells activation and maintenance by IL‐2. iNOS, inducible nitric oxide synthetase; NO, nitric oxide. (B) CD4^+^ T cell can interact with B cell by CD40L–CD40 interaction to mediate humoral immune response, such as the generation of memory B cells, the formation of GC and the generation of affinity‐matured and class‐switched plasma cells. In addition, Tfh‐derived IL‐21 also regulates GC B cell differentiation. (C) CD4^+^ T cells secrete IFN‐γ and TNF‐α to help reject tumors through damaging the formation of the vessel systems in tumor tissue. (D) Cytotoxic CD4^+^ T cells secrete IFN‐γ, granzymes, and perforin to mediate the direct tumor killing function.

In murine models of MHC‐II‐negative plasmacytoma, IFN‐γ secreted by CD4^+^ T cells can induce macrophage activation and M1‐polarization to prevent tumor progression (Figure 3A).^12^ M1‐like macrophages upregulate inducible nitric oxide synthetase (iNOS) and thereby subsequently increase tumor apoptosis triggered by nitric oxide (NO).^188^ In contrast with M1 macrophages, M2 macrophages activated by IL‐4 and IL‐13 mainly from Th2 cells^189^, ^190^ contribute to increasing the production of IL‐10 and arginase, resulting in the downregulation of NO production and restriction of the pathogen clearance ability.^191^, ^192^ Similarly, CD4^+^ T cell‐derived IFN‐γ mediates an increase in tumoricidal effectors, monocyte–macrophages, which secrete NO to induce remote inflammatory cell death in IFN‐unresponsive, MHC‐deficient tumor cells.^193^

Second, IL‐2 produced by CD4^+^ T cells has been proved to associated with the maintenance and the activation of NK cells,^194^, ^195^, ^196^ which could present antitumor effects through direct killing functions in murine models and clinical patients.^197^, ^198^

Additionally, it has been reported that CD4^+^ T cells play a vital role in the induction of humoral responses (Figure 3B). CD4^+^ T cell–B cell interaction relies on CD40–CD40L costimulatory signals, which mediate the generation of memory B cells and the formation of the GC,^199^, ^200^ which is positively associated with a favorable prognosis for a variety of cancers.^201^, ^202^, ^203^, ^204^, ^205^, ^206^, ^207^, ^208^, ^209^, ^210^, ^211^, ^212^, ^213^, ^214^ Moreover, CD40 signaling drives B cells into affinity‐matured and class‐switched plasma cells to modulate humoral immune responses against tumor^200^, ^215^ and multiple viral infections, including LCMV, HSV, and influenza virus infections.^216^, ^217^, ^218^ Similarly, ICOS plays an important role in the formation of a complete humoral response.^219^ In addition to these costimulatory signaling, Tfh‐derived IL‐21 also regulates GC B cell differentiation.^95^, ^215^

### 3.3 CD4^+^ T cells in antivessel formation

CD4^+^ T cells have been proven to inhibit tumor growth by inhibiting tumor blood vessel formation and maintenance (Figure 3C). According to some early studies, IFN‐γ can influence non‐bone marrow‐derived cells to limit the progression of tumors by inhibiting the blood vessel formation.^220^, ^221^ Even in tumor cells lacking of the expression of IFN‐γ receptors, IFN‐γ still helps prevent tumor growth.^220^, ^222^ In light of IFN‐γ‐deficient model and the phenomena observed from TNF‐deficient model using intravital microscopy, IFN‐γ induced vascular degeneration in the process of angiogenesis, inducing an ischemic state in the tumor tissue, while TNF‐α causes vascular rupture in the tumor tissue and promotes the infiltration of circulating cells into the TME when destroying blood vessels.^26^ Therefore, IFN‐γ and TNF‐α secreted by CD4^+^ T cells play a synergistic role in the inhibition of angiogenesis in tumor tissue by cutting off the blood supply to solid tumors to reduce tumor survival and metastasis.

### 3.4 Cytotoxic CD4^+^ T cells‐mediated antitumor effects

It is generally believed that the killing effects observed in antitumor immunity are mainly attributed to CTLs, while CD4^+^ T cells provide help and regulatory effects. However, many researchers have identified cytotoxic CD4^+^ T cells that display direct killing effects in autoimmune diseases, viral infections, and aging process.^223^, ^224^, ^225^, ^226^, ^227^, ^228^, ^229^, ^230^, ^231^, ^232^ In a murine virus infection model, CD4^+^ CTLs are distinguished by the expression of GZMB and their generation are inhibited by the Tfh‐related‐transcription factors BCL‐6 and TCF‐1.^233^ In addition, CD4^+^ CTLs lose the expression of THPOK and gain the expression of T‐BET and RUNX3 in intraepithelial lymphocytes,^229^, ^232^ whereas cytotoxic T‐cell‐like CD4^+^ T cells express EOMES in experimental autoimmune encephalomyelitis.^234^ According to another study on staphylococcal enterotoxin A, CD134 and CD137 costimulation programs the differentiation of cytotoxic Th1 cells, and Gzmb expression in cytotoxic Th1 cells depends on Eomes.^235^ Moreover, it has reported that CRTAM promotes CD4^+^ CTL differentiation by inducing the expression of Eomes and cytotoxic genes.^224^ Furthermore, the expression of Blimp‐1 greatly influences the cytotoxic function of CD4^+^ CTLs during influenza viral infections.^236^

Similarly, in a murine tumor model, CD4^+^ T cells can also be differentiated into cytotoxic CD4^+^ T cells with the secreting of IFN‐γ, granzymes, and perforin (Figure 3D).^14^, ^237^ Moreover, tumor regression mediated by cytotoxic CD4^+^ T cells is dependent on MHC‐II‐restricted manner, which could be enhanced by anti‐CTLA‐4 antibody.^238^ The existence and function of cytotoxic CD4^+^ T cells that express cytolytic effector molecules, such as GZM (e.g., GZMA, GZMB, GZMH, GZMK), PRF1, NKG7, and GNLY are also supported by single‐cell sequencing results in human tumors.^239^, ^240^, ^241^, ^242^ According to the results of single‐cell sequencing in human bladder cancer, cytotoxic CD4^+^ T cells are also polyfunctional due to the expression of GZMA, GZMB, GZMK, PRF1, GNLY, and NKG7.^242^ Such cytotoxic CD4^+^ T cells directly kill autologous tumors in a MHC‐II‐dependent manner in response to immunotherapy.^242^ However, the function of cytotoxic CD4^+^ T cells could be diminished by autologous Tregs.^242^, ^243^ Interestingly, cytotoxic activity is partly dependent on SLAMF‐7 and enhanced by the combined application of SLAMF‐7 agonist.^241^ In general, the killing function of cytotoxic CD4^+^ T cells depends on MHC‐II and the expression of cytolytic effector molecules.

The expression of transcription factors in the cytotoxic CD4^+^ T cell subset in TME is various according to the type of tumors. In a melanoma model treated with adoptive cell transfer (ACT), Th1 cytotoxic T cells differentiated from naïve CD4^+^ T cells secreted granzyme B, perforin and IFN‐γ, and expressed the transcription factor T‐bet.^237^ In contrast, in adoptive transfer model bearing melanoma, Gzmb^+^CD4^+^ T cells generated under the additional use of anti‐OX40 agonists (OX86) and cyclophosphamide (CTX) were also Eomes^+^. Compared with Eomes knockdown antigen‐specific cells, this combined treatment enhanced the killing function of CD4^+^ CTLs according to the required expression of Eomes.^244^ In a model of enhanced CD4^+^ T cell cytotoxic function through CTLA‐4 blockade, a favorable effect was involved in the competition of IL‐2 binding with Treg cells, and a specific gene‐deficient model demonstrated that the expression of GZMB in CD4^+^ CTLs induced by IL‐2 depended on neither EOMES nor T‐BET, but instead relied on BLIMP‐1,^243^ which limited the expression of BCL‐6 and TCF‐7.^88^, ^245^, ^246^, ^247^ Notably, the robust transcriptional map of CD4^+^ CTL generated from single‐cell RNA sequencing in patients with melanoma consistently indicates that, compared with Th cells, cytotoxic CD4^+^ T cells enrich the expression of RUNX3, unlike T‐BET, EOMES and BLIMP‐1.^241^

With the IL‐12 inducing production of granzymes and perforin through the STAT4 pathway, Th1 cells acquire cytotoxic functions,^224^, ^248^ while CD4^+^ CTLs can also be generated from various cells, such as Th0, Th1, and Th2, under the action of IL‐2 and IL‐12 in certain conditions. Among these, IL‐2 plays an important role in inducing the differentiation into CD4^+^ CTLs with improved cytotoxic functions, especially Th0.^224^, ^248^

## 4 CD4^+^ T CELL ROLES IN PROTUMOR AND PROCHRONIC VIRAL RESPONSES

In addition to antitumor and antichronic viral responses of CD4^+^ T cells, CD4^+^ T cells also play a role in protumor and prochronic viral responses. The subpopulation of CD4^+^ T cells, Treg cells, can mediate an immunosuppression to inhibit effector T cells function, thereby inducing tumor progression and chronic viral persistence. Moreover, exhausted CD4^+^ T cells exhibit reduced effector function and proliferation and increased expression of inhibitory receptors, which can promote protumor and prochronic viral responses.

### 4.1 Immunosuppression mediated by Treg cells

In contrast to other CD4^+^ T cell subpopulations, Treg cells mainly mediate immunosuppression and immune tolerance.^249^, ^250^, ^251^, ^252^, ^253^ The dominant feature of Treg cells is the expression of FOXP3, CD25, and CTLA‐4, which helps maintain immunosuppression.^114^, ^115^, ^116^, ^117^ According to a transcriptome analysis based on human cancers, tumor‐infiltrating Treg cells also upregulate the expression of some immunosuppressive molecules, such as LAG‐3, TIM‐3, T cell immunoreceptor with Ig and ITIM domains (TIGIT), CTLA‐4,^254^, ^255^ and some molecules involved in activation, including ICOS, CD137, OX40, and GITR.^256^ In addition, TCF‐1‐deficient Treg cells could significantly inhibit T cell proliferation and cytotoxicity to promote tumor growth. In colorectal cancer patients, tumor‐infiltrating Treg cells expressed lower levels of TCF‐1 compared with those in the blood.^257^ According to clinical studies, the degree of Treg cell infiltration correlates with poor prognosis for many human cancers, such as breast, ovarian, gastric, and colorectal cancer.^258^, ^259^, ^260^, ^261^, ^262^ Notably, two subsets of FOXP3^+^ T cells are associated with different outcomes in colorectal cancer. The FOXP3^hi^ subset is primarily immunosuppressive cells associated with poor prognosis, whereas the FOXP3^lo^ subset is considered non‐Treg cells, which mainly produce inflammatory cytokines leading to better prognosis.^263^

#### 4.1.1 Immunosuppression mediated by Treg cells in tumors

The immunosuppressive effect induced by Treg cells was first proposed in studies on tumor rejection by using anti‐CD25 to delete CD25^+^CD4^+^ Treg cells.^264^, ^265^ In TME, Treg cells induce immunosuppressive effects, which predominantly maintained by TCR signaling^266^ to inhibit tumor regression by diverse pathways ^267^, ^268^ In addition to TCR signaling, CD25 can induce STAT5 activation to maintain the suppressive function of Treg cells. CD25, also known as IL‐2Rα, cooperating with IL‐2R β and γ chains form high‐affinity IL‐2R complex to competitively bind to IL‐2. The competitive depletion of IL‐2 prevents further activation of effector T cells and inhibits maintenance of the T cell pool as a consequence of the weak immune response.^269^ Moreover, Treg cells also express CTLA‐4 costimulatory receptors with a higher affinity to competitively binds to the CD80/CD86 on the surface of DCs than CD28, thereby downregulating the CD28–CD80/CD86 costimulation signal, which is required in the second step of T cell activation.^121^, ^122^, ^123^ Another study demonstrated that the trans‐endocytosis of CD80/CD86 occurred between APCs and Treg cells when interacting with CTLA‐4, leading to the downregulation of CD80/CD86.^122^ In addition, the interaction of CTLA‐4 with CD80/CD86 promotes the production of indoleamine 2,3‐dioxygenase (IDO), which is an essential enzyme that degrades tryptophan and subsequently promotes a lack of tryptophan in the TME, resulting in the inhibition of T cell proliferation.^270^, ^271^ In a mouse model, CTLA‐4‐deficiency inhibited the immunosuppressive effects of Treg cells, specifically, the Treg‐mediated downregulation of CD80/CD86 expression on DCs, and enhanced the antitumor response.^123^ Consistently, it has been reported that the response to CTLA‐4 blockade mainly depends on a dramatic decrease in Treg cells.^272^, ^273^, ^274^ In addition, the clinical efficacy of the monoclonal antibody ipilimumab (anti‐CTLA‐4) in patients with clinical melanoma is associated with selective depletion of Treg cells based on the FcγR‐dependent mechanism and an increase in the tumor‐infiltrating CD8^+^ T/Treg cell and Teff/Treg ratios.^275^ In addition to these mechanisms, Treg cells can also inhibit antitumor immunity by secreting cytokines, such as TGF‐β, IL‐10, and IL‐35, which contribute to an immunosuppressive effect in TME.^276^, ^277^, ^278^

#### 4.1.2 Immunosuppression mediated by Treg cells during chronic viral infection

CD4^+^CD25^+^ Treg cells increase in HBV‐infected patients during chronic HBV infection^279^, ^280^, ^281^ and mediate decreased proliferation of peripheral blood mononuclear cells (PBMC).^279^, ^281^ Furthermore, the depletion of CD4^+^CD25^+^ Treg cells can elevate HBV‐specific T cells responses.^279^, ^280^ A decline of PBMC proliferation and IFN‐γ production in HBV individuals are observed by restoring depleted CD4^+^CD25^+^ Treg cells.^279^ Notably, viral load is correlated with the frequency of circulating CD4^+^CD25^+^ Treg cells.^281^, ^282^ Moreover, Treg cells can mediate an immunosuppressive effect and a defect of immune response to HBV virus.^279^

In addition, at the initial time of HCV infection, the Foxp3 expression and Treg cells immunosuppression are similar in patients with resolved HCV infection spontaneously or persistence HCV infection.^283^ However, during HCV persistence, the upregulation of Treg cells has been observed, which directly inhibits HCV‐specific CD8^+^ T cell responses^284^ by curtailing the proliferation and perforin expression.^285^ Similarly, one study also demonstrated that CD4^+^CD25^+^ Treg cells attenuated antigen‐specific proliferation and IFN‐γ production in antigen‐specific CD8^+^ T cells during persistent HCV infection.^286^ Furthermore, the depletion of CD4^+^CD25^+^ Treg cells mediates an increase in the peptide‐specific expansion and the number of IFN‐γ^+^CD8^+^ T cells^285^ during persistent HCV virus infection. However, the impairment induced by CD4^+^CD25^+^ Treg cells is not in antigen‐specific manner or limited to HCV‐specific CD8^+^ T cells, but also applies to influenza virus‐specific CD8^+^ T cells.^286^ In addition to the suppression of antigen‐specific proliferation and IFN‐γ production in HCV‐specific T cells, CD4^+^CD25^+^ Treg cells can impair activation‐induced cell death mediated by HCV‐specific T cells.^287^

### 4.2 CD4^+^ T cell exhaustion

**FIGURE 4:**
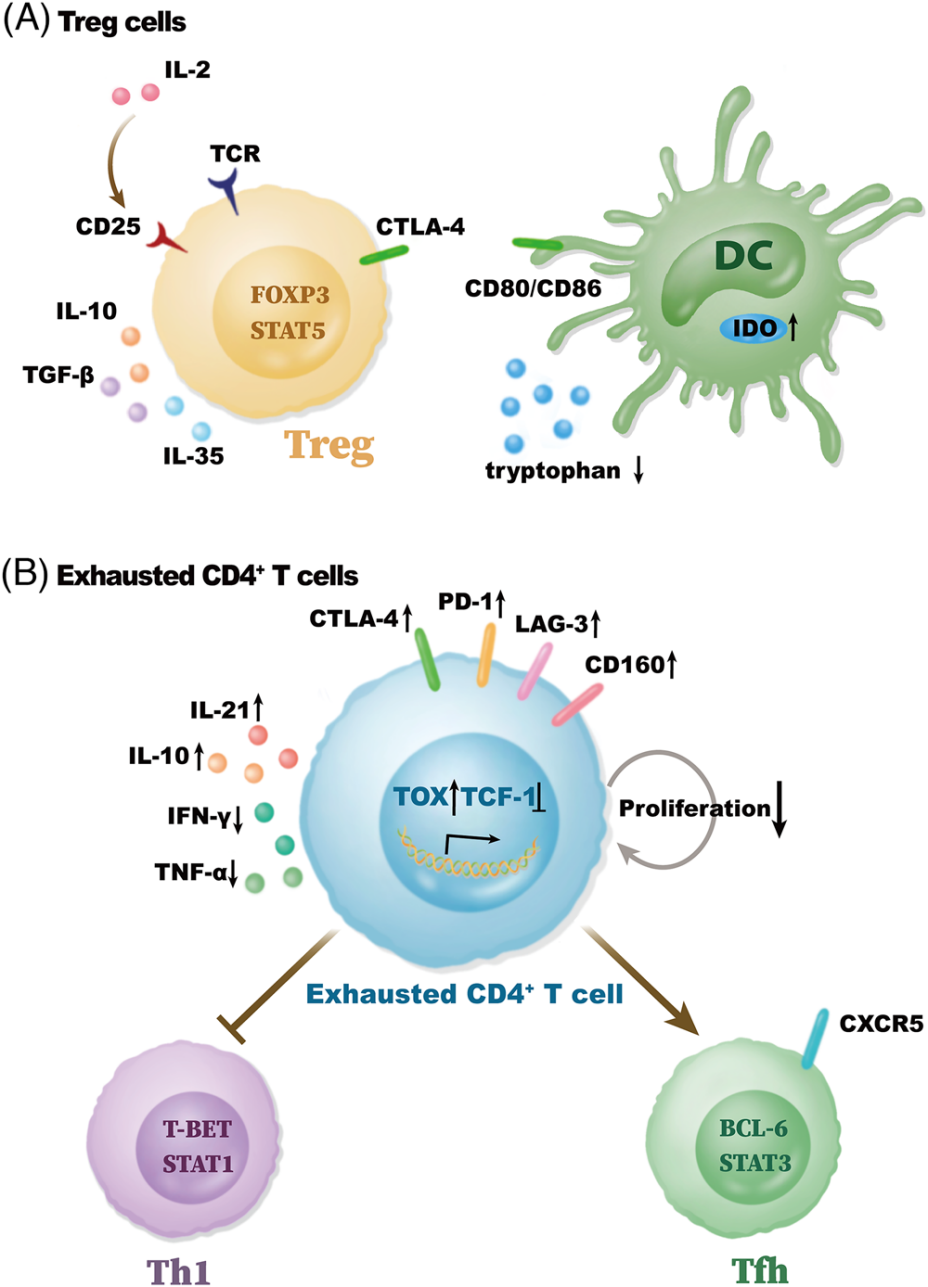
Treg cells and exhausted CD4^+^ T cells in protumor and prochronical viral infection. (A) Treg cells can mediate immunosuppressive functions through various ways, including CD25, CTLA‐4, IL‐10, TGF‐β, and IL‐35. CD25 cooperating with IL‐2R β and γ chains can competitively binding to IL‐2 to inhibit activation and maintenance of effector T cells. In addition, CTLA‐4 on Treg cells contributes to decreasing the CD28‐CD80/CD86 signal on DCs and increasing the secretion of IDO from DCs, inducing a lack of tryptophan in TME and subsequently inhibiting T cell proliferation. IDO, indoleamine 2,3‐dioxygenase; IL‐2R, IL‐2 receptor. (B) Exhausted CD4^+^ T cells exhibit upregulation of TOX and inhibitory receptors, including PD‐1, CTLA‐4, LAG‐3, and CD160, while lose the expression of SLAMF‐6 and TCF‐1. Additionally, exhausted CD4^+^ T cells mediate a reduction of proliferation and function with decreased secretion of IFN‐γ and TNF‐α. However, IL‐21 and IL‐10 are significantly upregulated. Furthermore, CD4^+^ T cells exhibit a loss of Th1 cells and an enrichment of Tfh cells.

Owing to continued antigen stimulation, CD8^+^ T cells become exhausted in tumors and chronic viral infections. Similar to the CD8^+^ T cells, CD4^+^ T cells also exhibit an exhaustion status in tumors and chronic viral infections. Meanwhile, exhausted CD4^+^ T cells exhibit reduced effector function and proliferation, altered transcriptional expression, and increased expression of inhibitory receptors, such as CTLA‐4, PD‐1, LAG‐3, and CD160 (Figure 4).

#### 4.2.1 Exhausted CD4^+^ T cells in tumors

In multiple types of tumors, the evidence of CD4^+^ T cells expressing inhibitory receptors (PD‐1, TIM‐3, CTLA‐4, and LAG‐3) suggests that CD4^+^ T cells become exhausted.^288^, ^289^, ^290^ In a melanoma tumor model, the adoptive transfer of tumor antigen‐specific CD4^+^ T cells can prevent tumor progression. However, some of the mice experience tumor relapse.^291^, ^292^ Tumor‐infiltrating CD4^+^ T cells in recurrent tumors express higher levels of inhibitory receptors and have a lower capacity of cytokine production.^292^ Compared with peripheral blood derived CD4^+^ T cells^293^, ^294^ and nonmalignant infiltrating CD4^+^ T cells,^295^, ^296^, ^297^ tumor‐infiltrating CD4^+^ T cells express high levels of inhibitory receptors.^298^ This indicates that continuous antigen stimulation can induce exhaustion of CD4^+^ T cells. In addition, the expression levels of inhibitory receptors on CD4^+^ T cells seem to be associated with tumor progression and clinical stage.^299^, ^300^ Higher expression levels of inhibitory receptors indicate advanced tumor progression; however, a reduction in inhibitory receptors is related to tumor regression.^301^, ^302^, ^303^, ^304^ In addition to inhibitory receptors, CD4^+^ T cells in TME exhibited poor effector function in a murine melanoma model.^305^ In human patients, CD4^+^ T cells in tumors also exhibit reduced proliferation and cytokine production.^306^, ^307^ Nevertheless, ICB can partially reinvigorate the function of CD4^+^ T cells.^295^, ^299^, ^307^, ^308^ In addition, the combination of PD‐L1 and LAG‐3 blockade downregulates the expression levels of inhibitory receptors of CD4^+^ T cells.^292^, ^309^

TCF‐1 is a key transcription factor for the progenitor exhausted CD8^+^ T cells.^310^ In addition to TCF‐1, Wherry et al. defined SLAMF6 as an important marker of the progenitor exhausted CD8^+^ T cells.^311^ Progenitor exhausted CD8^+^ T cells can self‐renew and give rise to terminal exhausted CD8^+^ T cells.^312^, ^313^, ^314^ Compared with CD4^+^ T cells in spleen, tumor‐infiltrating CD4^+^ T cells lost expression levels of TCF‐1 and SLAMF6 in a melanoma model, indicating that tumor‐infiltrating CD4^+^ T cells seem to be a transitory or terminal exhausted T cells.^315^ When treated with a PD‐L1 blockade, tumor‐infiltrating CD4^+^ T cells upregulate TCF‐1 and downregulate TIM‐3 and LAG‐3.^315^ CD39 has been reported as a marker of CD8^+^ T cell exhaustion, with CD39^high^CD8^+^ T cells showing reduced capacity of cytokine production.^316^ Tumor‐infiltrating CD4^+^ T cells also express CD39, which express higher levels of PD‐1 and produce fewer cytokines.^317^ After PD‐1 blockade, CD4^+^ T cells have been reported to produce more cytokines.^317^ Moreover, PD‐1 blockade enhances DC maturation and CD8^+^ T cells proliferation.^317^ TOX can drive the epigenetic programming of exhausted CD8^+^ T cells^318^, ^319^, ^320^ with the highest expression being exhibited by terminal exhaustion T cells compared with other exhaustion subsets.^321^ Similar to tumor‐infiltrating CD8^+^ T cells, those CD39^+^CD4^+^ T cells also express TOX.^317^

#### 4.2.2 Exhausted CD4^+^ T cells in chronic viral infection

CD4^+^ T cells are unessential for controlling acute viral infections, while they play an important role in the elimination of chronic viral infection due to their roles of helping CD8^+^ T cells priming and sustaining their function. Similar to CD8^+^ T cells, CD4^+^ T cells become exhausted due to continued antigen stimulation during chronic viral infection. Nonetheless, exhausted CD4^+^ T cells received less attention and was less known about their mechanism of action during chronic viral infection. Compared with LCMV Armstrong infection, during the early stage post LCMV clone‐13 infection, continued viral antigen stimulation mediates the functional inactivation of virus‐specific CD4^+^ T cells with losing the ability of producing effector cytokines, such as IFN‐γ and TNF‐α.^143^ Despite the decline in IFN‐γ, the IFN signaling is sustained in virus‐specific T cells,^322^, ^323^ which in turn modulates an increase in the ISG signature. In addition, IL‐21, which is critical for CD8^+^ T cells and B cells, increases during chronic viral infections as compared with acute viral infections.^175^, ^324^ Moreover, IL‐10, which suppresses T cells function, also drastically upregulates during LCMV clone‐13 infection^325^, ^326^ and HIV infections.^327^, ^328^

In acute HCV infection, the vigorous proliferation of virus‐specific CD4^+^ T cells in blood is correlated with the elimination of HCV virus.^329^ Moreover, multiepitope‐specific CD4^+^ T cells are detected at an early stage of HCV infection, suggesting a broad response of CD4^+^ T cells in HCV‐infected individuals.^329^ The breadth of the response and the repertoire of targeted epitopes are not significantly different between acute spontaneously resolving (AR) individuals and acute chronically evolving (AC) HCV‐individuals.^329^ Nevertheless, consistent with the exhausted CD8^+^ T cells, the proliferative capability of exhausted CD4^+^ T cells is impaired during chronic viral infection.^330^, ^331^ The early reduction in proliferative capability of HCV‐specific CD4^+^ T cells is correlated with persistent viremia.^329^ Meanwhile, virus‐specific CD4^+^ T cells progressively decreased due to persistent viral antigens.^329^

The alterations observed in exhausted CD4^+^ T cells include not only the loss of poly‐functionality and proliferative capability, but also the expression of multiple inhibitory ligands. Virus‐specific CD4^+^ T cells upregulate PD‐1 expression and the expression level of PD‐1 is correlated with viral load and disease stage.^332^, ^333^, ^334^, ^335^ In addition, PD‐1 expression in HIV‐specific T cells is significantly higher in the lymph node than in the blood.^332^ Consistent with PD‐1 expression, the expression level of CTLA‐4 also increases in virus‐specific CD4^+^ T cells during HIV infection and is positively correlated with disease progression.^334^, ^336^ Moreover, Tim‐3 is also coexpressed with PD‐1 and CTLA‐4 in virus‐specific CD4^+^ T cells during HIV infection.^337^ In addition to these inhibitory molecules, virus‐specific CD4^+^ T cells also coexpress other inhibitory ligands during LCMV clone‐13 infection, including CD160 and LAG‐3.^338^ Similarly, CD4^+^ T cells express high level of PD‐1 and LAG‐3 during chronic HBV infection.^339^

During chronic viral infection, Th1 cells progressively lose, while virus‐specific CD4^+^ T cells tend to polarize into Tfh cells.^340^, ^341^, ^342^ At the early time of viral infection, CXCR5^+^SMATA cells equally upregulate in both acute infection and chronic infection.^341^ Strikingly, due to persistent antigen stimulation during chronic infection, CXCR5^+^SMATA cells, which are referred to as Tfh cells, dramatically increase.^341^ In contrast, the frequency of CXCR5^+^SMATA cells are comparable at different periods of acute viral infection.^341^ Sustained antigen or TCR stimulation maintains the expression of Tfh‐associated transcription factors, such as CXCR5 expression.^341^, ^343^ Moreover, master transcription factors in virus‐specific Tfh cells also increase during chronic infection, including ICOS and Bcl‐6.^341^ Heterogeneous transcription factor profiles of exhausted CD4^+^ T cells confirm a loss of Th1 cells and an enrichment of Tfh cells during chronic viral infection.^340^ Similar to chronic LCMV infection, Tfh cells also increase in HIV‐infected individuals.^344^ Moreover, an accumulated population of Tfh cells exhibit an uncomparable transcriptional profiles in SIV‐infected tissues compared with SIV‐negative tissues.

Tfh cells are critical for B cells to produce class‐switched and high‐affinity antibodies. Neutralizing antibodies are generated in acute infection; however, they are impaired in Tfh cells during chronic viral infection.^345^ In contrast, non‐neutralizing antibodies are enhanced^345^ during chronic infection. Furthermore, polyclonal hypergammaglobulinemia is exhibited in chronic virus‐infected mice^345^, ^346^ and is associated with the expression level of BCL‐6 in HIV‐Tfh cells.^346^ During chronic hepatitis C infection, vaccine responses toward hepatitis B virus are impaired and CD4^+^ T cell responses are downregulated in vaccine nonresponders compared with those in vaccine responders, indicating the defect of Tfh‐B cells interaction and a poor ability of Tfh cells to help B cells.^347^ Similarly, Tfh cells have an inability to relay help to B cells in HIV‐infected individuals.^344^ During HIV and SIV infection, Tfh cells constitute a major reservoir of HIV virus to infect, replicate and produce.^348^, ^349^, ^350^ The HIV viral load is correlated with the percentage of Tfh cells.^350^ Moreover, Tfh cells are a restrict compartment for SIV virus and highly reside in B cell follicles of lymph nodes.^350^ SIV‐specific CD8^+^ T cells can only mediate the clearance of SIV virus at extra‐follicular sites. Hence, SIV virus cannot be cleared in virus‐infected Tfh cells,^348^ suggesting that Tfh cells can prevent SIV virus from being eliminated by SIV‐specific CD8^+^ T cells.

## 5 CD4^+^ T CELL‐BASED IMMUNOTHERAPIES

Immunotherapies, including ACT therapy, vaccines, and ICB, have revolutionized cancer and chronic viral treatments. Most of these immunotherapies are mainly focused on CD8^+^ T cells; however, CD4^+^ T cells‐based immunotherapies recently receive more attention in tumors and chronic viral infections due to the crucial roles of CD4^+^ T cells. Notably, some of the CD4^+^ T cells‐based immunotherapies have already been approved, such as CTLA‐4. All of the clinical trials of the immunotherapies based on CD4^+^ T cells are listed in Table 1.

**TABLE 1:** Clinical trials of the immunotherapies based on CD4^+^ T cells.

Type	Cancer	Phase	Trial number	Clinical outcome	References
ACT	Metastatic digestive tract cancers	Phase II	NCT01174121	–	^351^
	Glioblastoma	Phase I	NCT02208362	Ongoing	^352^
	Relapsed or refractory chronic lymphocytic leukemia, non‐Hodgkin lymphoma*, or acute lymphoblastic leukemia	Phase I/II	NCT01865617	Best overall response rate: 51% Complete remission: 40% Median PFS of complete remission patients: 20.0 months	^353^, ^354^
	B‐cell leukemia* or lymphoma	Phase I	NCT01029366	Complete remission: 90% 6‐month event‐free survival rate: 67% OS: 78%	^355^, ^356^
Vaccine	Prostate cancer	Phase I	EudraCT 2006‐ 003299‐37	No beyond grade 2 Toxicities	^357^
	Prostate cancer	Phase I/II	NCT03199872	No treatment‐related adverse events of grade ≥3	^358^
	Noninvasive vulvar/vaginal lesions	Phase I/II	NL21215.000.08	Complete histologic response: 35%	^359^
	Adenocarcinoma of the pancreas	Phase I/II	CTN RAS 95002	Median survival: 148 days (Immune responders)	^360^
	Non‐small cell lung cancer (NSCLC)	Phase II	CTN‐2006	Median PFS: 371 days (Immune responders)	^361^
	Prostate and renal cancers	Phase I/II	EudraCT number 2009‐011330‐10	Median OS: 369 days	^362^
	Glioblastoma	Phase II	NCT04280848	Ongoing	–
	Metastatic non‐small cell lung cancer	Phase I/II	NCT02818426	The 1‐year PFS: 17.2% Median OS:11.6 months (Immune responders)	^363^
	Glioblastoma	Phase I	NCT02149225	Median OS: 29.0 months Median PFS: 14.2 months	^364^
	Metastatic melanoma	Phase II	NCT00071981	Median OS: 11.8 months	^365^
	HER2^pos^ DCIS	Phase I/II	NCT02061332	Pathologic complete response:13/54	^366^
ICB	Advanced melanoma	Phase I	NCT00920907	–	^367^
	Metastatic castration‐resistant prostate cancer	Phase II	NCT02703623	Ongoing	^368^
Vaccine + ICB	Human papillomavirus (HPV) positive cancers	Phase II	NCT03946358	Ongoing	^369^
	Non‐small cell lung cancer	Phase II	NCT04263051	Ongoing	–
Other therapy	Advanced solid tumors	Phase I	NCT01460134	Treatment‐related adverse events: grade 1/2	^370^
	Advanced or metastatic carcinoma, melanoma, or non‐small cell lung carcinoma	Phase I	NCT01295827	For non‐small cell lung carcinoma, Median OS in treatment‐naive patients: 22.3 months Median OS in previously treated patients: 10.5 months	^371^, ^372^

ACT, adoptive cell transfer; ICB, immune checkpoint blockade; Ref, reference; PFS, progression‐free survival; OS, overall survival; *, clinical outcome only in this cancer.

### 5.1 Adoptive cell transfer

**FIGURE 5:**
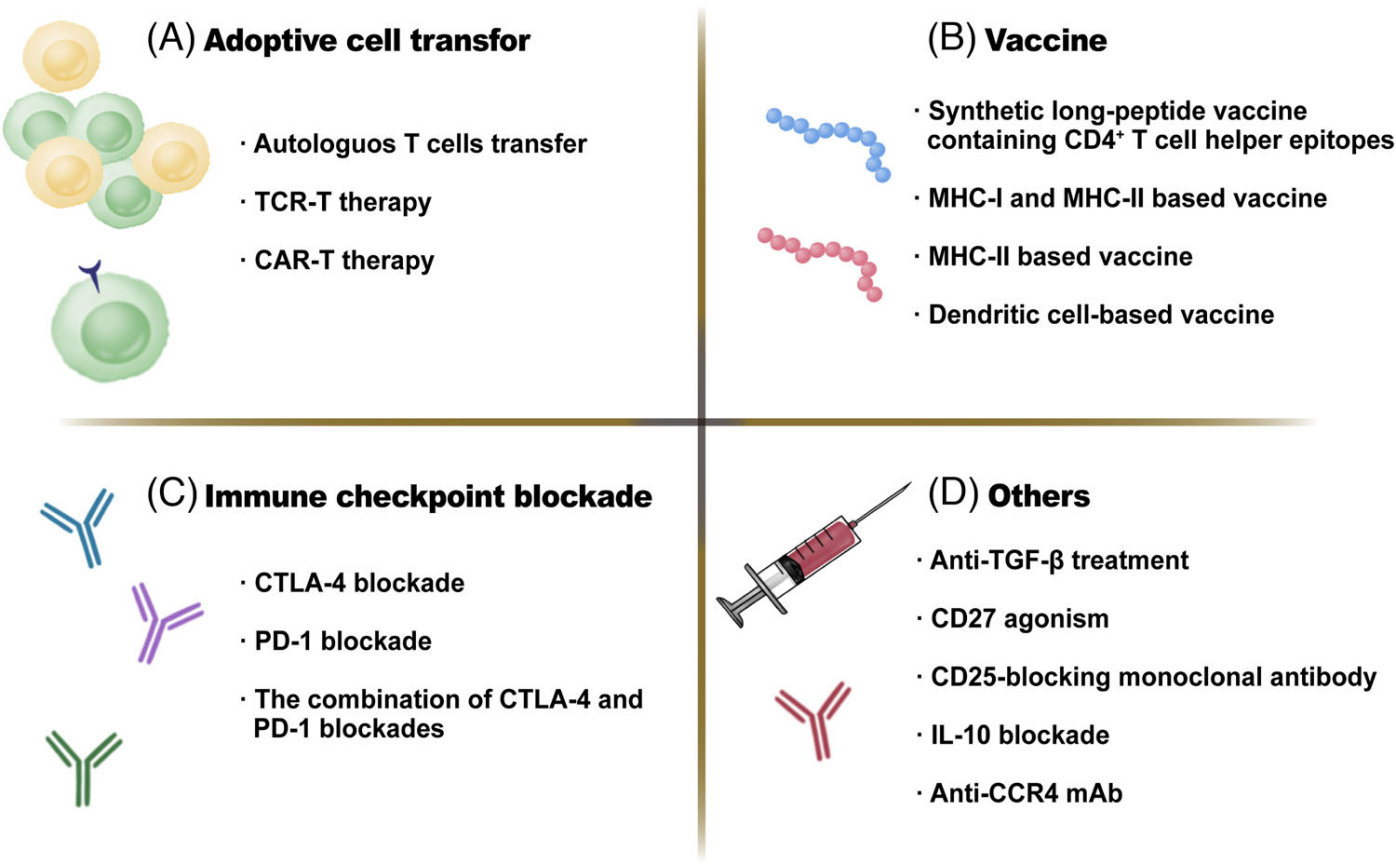
Immunotherapies based on CD4^+^ T cells. (A) Adoptive cell transfer (ACT) therapy includes autologous CD4^+^ T cells transfer, CD4^+^ chimeric antigen receptor (CAR) T cell transfer, and engineered CD4^+^ T cell receptor (TCR) T cell transfer. (B) Vaccines. MHC‐II‐restricted epitope‐based vaccine strategy, MHC‐I‐restricted epitope and MHC‐II‐restricted epitope‐based vaccine strategy, synthetic long‐peptide (SLP) containing helper epitope vaccine strategy, and dendritic cell‐based vaccines are available. (C) Immune checkpoint blockade aims to inhibit immune checkpoint receptors axis, including CTLA‐4 and PD‐1. (D) Other treatments. Considering the important role of cytokines and costimulatory receptors, there are some other strategies, such as anti‐TGF‐β and CD27 agonism and so on.

ACT therapy mainly involves the use of natural tumor‐reactive lymphocytes, genetically engineered lymphocytes expressing chimeric antigen receptors (CARs) or TCRs (Figure 5A). Most of the ACT therapies focus on CD8^+^ T cells instead of CD4^+^ T cells due to the robust effector function of CD8^+^ T cells. However, the transfer of autologous NY‐ESO‐1‐specific CD4^+^ T cells can induce a durable clinical remission in patients with metastatic melanoma.^373^ Moreover, ACT with approximately 25% mutation‐specific Th1 cells was shown to mediate tumor regression in patients with metastatic epithelial cancer.^351^ Upon tumor progression, the patients were re‐transferred with >95% pure mutation‐specific Th1 cells, which effectively suppressed the tumor volume,^351^ indicating that CD4^+^ T cells can also be harnessed for ACT treatment. During the CAR‐T therapy for patients with myeloma, a higher CD4/CD8 ratio is associated with a better clinical response.^374^, ^375^ Moreover, the administration of CD4^+^ M28z CAR‐T cells alone for mesothelioma is efficacious and contributes to tumor suppression and the administration of CD4^+^19z1^+^ T cells alone for B cell tumors could prolong the survival of tumor‐bearing mice.^376^, ^377^ In accordance with previous studies, the adoptive treatment with MHC‐II‐restricted and MAGE‐A3‐specific CAR‐T cells is efficacious to generate tumor eradication, which highlights the importance of CD4^+^ T cells in ACT therapy.^378^

Owing to continuous antigen stimulation, the transferred autologous CD8^+^ T cells, CD8^+^ CAR‐T cells,^352^, ^379^, ^380^ or CD8^+^ TCR‐T cells^381^, ^382^ become rapidly exhausted and loss the capacity of effector function. One study reported that, in contrast to CD8^+^ CAR‐T cells, glioblastoma‐targeted CD4^+^ CAR‐T cells could maintain the T cell pool and retain its effector function.^352^ Consistent with the report, CD4^+^ CAR‐T cells can sustain efficacy in vivo compared to CD8^+^ CAR‐T cells.^382^ Meanwhile, CD4^+^ CAR‐T cells also exhibit equivalent cytotoxicity with CD8^+^ CAR‐T cells.^382^ Moreover, administration of the CD4‐targeted lentiviral vector to generate CAR‐T cells induces a better killing function than administration of the CD8‐targetd lentiviral vector.^383^ Surprisingly, CD4^+^ CAR‐T cells are slightly more polyfunctional than CD8^+^ CAR‐T cells and have a highly mixed Th1/Th2 function.^47^ Moreover, TCR engineered BRAFV600E‐specific CD4^+^ T cells have been reported to enhance the CD8^+^ T cell response in patients with melanoma and induce an antitumor effect.^384^ Nevertheless, CD4^+^ CAR‐T cells efficiently eliminate tumors, and the combination of CD4^+^ and CD8^+^ CAR‐T cells potentiates antitumor activity.^353^, ^354^, ^385^ Furthermore, T‐bet can improve the antitumor effect of CD4^+^ CAR‐T cells against B7H6‐expressing tumor cells by upregulating the secretion of cytokines and cytotoxicity. Recently, long‐persisting CD4^+^CD19^+^ CAR‐T cells have been reported to emerge in patients with chronic lymphocytic leukemia who achieved complete remission.^355^, ^356^ Such long‐persisting CD4^+^CD19^+^ CAR‐T cells display cytotoxicity, activation, and proliferation.^355^

### 5.2 Vaccines

To exploit the cytotoxic capacity of CD8^+^ T cells, most of the vaccines have been designed using MHC‐I‐restricted epitopes.^386^, ^387^ However, some of the CD8^+^ T cell‐based vaccine strategies do not induce favorable clinical outcomes. For example, a CD8^+^ T cell‐based vaccine strategy without CD4^+^ T cell help cannot induce the formation of cytotoxicity in CD8^+^ T cells.^77^ Besides, a synthetic long‐peptide (SLP) vaccine predicted to bind MHC class I raised CD4^+^ T cell responses.^388^ Similarly, despite the absence of MHC‐II binding prediction, a synthetic RNA mutanome vaccine for MHC‐I‐restricted prediction has also been reported to elicit CD4^+^ T cell responses.^389^ Likewise, neoantigen vaccines with MHC‐I binding affinity also yield a CD4^+^ T cell response,^390^, ^391^ suggesting that the generation of polyfunctional CD8^+^ T cell responses is associated with CD4^+^ T cell help. In addition, continuous MHC‐I‐restricted epitope stimulation promotes CD8^+^ T cell exhaustion.^392^, ^393^ Therefore, MHC‐II‐restricted epitope‐based vaccine strategy has been utilized (Figure 5B) and the clinical trials of these vaccines are listed in Table 1. In patients with prostate cancer, a SLP vaccine containing CD4^+^ T cell helper epitopes can induce both CD4^+^ and CD8^+^ T cell responses.^357^, ^358^ Similar observations have been reported for a SLP vaccine with the inclusion of a helper epitope in patients with human papillomavirus (HPV)‐induced intraepithelial neoplasia.^359^, ^394^ In patients with pancreatic cancer, a mutated Ras‐SLP was aimed to predominately activate CD4^+^ T cells can be synergized with GM‐CSF and mediate a robust CD4^+^ T cell response to prolong survival,^360^, ^395^ underlining the crucial role of MHC‐II‐restricted epitopes in immunotherapies.

Human telomerase reverse transcriptase (h‐TERT) is expressed in multiple types of cancer, including non‐small‐cell lung cancer (NSCLC). Several studies have suggested that the h‐TERT vaccine incorporating helper epitopes drives favorable clinical outcomes in patients with lung, prostate, renal, and pancreatic cancer.^361^, ^362^, ^396^, ^397^ Concomitantly, the vaccines based on HLA‐II binding h‐TERT derived epitopes referred as universal cancer peptides (UCP) elicit Th1 and CD8^+^ T cell responses and promote the infiltration of h‐TERT‐specific CD8^+^ T cells,^398^, ^399^ confirming that MHC‐II‐restricted epitope‐based vaccines can mediate potent antitumor effects. This UCP‐based vaccine was evaluated on patients with glioblastoma (NCT04280848) and metastatic NSCLC^363^ in clinical trials. UCP‐based vaccine combined with ICB was also evaluated in individuals with HPV positive cancers^369^ and NSCLC (NCT04263051). Moreover, MHC‐II epitopes can shape antitumor immunity and synergize with immunotherapy.^400^, ^401^ In our study, we found that the MHC‐II‐restricted epitope‐based heterologous prime‐boost vaccination potentiates antitumor immunity and PD‐1/PD‐L1 immunotherapy.^402^ Besides, a heterologous prime‐boost immunization based on MHC‐II‐restricted epitope selectively induces virus‐specific CD4^+^ T cell responses and control chronic viral infection.^403^ To attempt to take advantage of both CD8^+^ T cells and CD4^+^ T cells, MHC‐I and MHC‐II‐based vaccination was carried out and displayed strong immunogenicity.^364^, ^365^ Since DCs delivery CD4^+^ T cell help to CD8^+^ T cells, several studies have initiated DC‐based vaccine pulsed by the HER2 MHC‐II epitopes^366^, ^404^, ^405^ and these vaccine strategies mediate tumor elimination.^366^, ^405^

### 5.3 Immune checkpoint blockade

Similar observation in CD8^+^ T cell response to ICB, CD4^+^ T cells have also been reported to be required for ICB (Figure 5C), especially CTLA‐4 blockade in tumor patients.^406^, ^407^, ^408^ Likewise, the positive clinical outcome reflects an enhanced percentage of CD4^+^ T cells in patients treated with ipilimumab,^409^ indicating that CD4^+^ T cells play a crucial role in the ICB therapies.^410^ In addition, the repertoire of systemic CD4^+^ T cells prior to immunotherapy correlates with the antitumor efficacy of CTLA‐4 or PD‐1 blockade therapy.^411^ Moreover, CD4^+^CD62L^low^ T cells in the blood prior to immunotherapy are correlated with the response to PD‐1 blockade,^412^ highlighting the critical contribution of CD4^+^ T cells. The efficacy of CTLA‐4 blockade is due to the tumor‐infiltrating Treg cell depletion or reduction.^273^, ^274^ Interestingly, the combination of CTLA‐4 and PD‐1 blockade increases the percentage of Th1‐like CD4^+^ T cells.^413^ In addition, CTLA‐4 blockade mediates ICOS^+^ (or ICOS^hi^) Th1‐like CD4^+^ T cell expansion.^31^, ^414^ Furthermore, CD44^+^PD‐1^−^CD127^low^CD4^+^ T cells, referred as unexhausted cells, significantly increase in responders treated with ICB,^415^ demonstrating that unexhausted CD4^+^ T cells expand to restrain tumor progression in response to ICB treatment. In addition, tumor‐specific cytotoxic CD4^+^ T cells express lytic granules induced by Eomes after ipilimumab treatment in patients with advanced melanoma.^367^

PD‐1/PD‐L1 blockade can restore the function and proliferation of HIV‐specific CD4^+^ T cells.^332^, ^333^, ^334^, ^335^ Moreover, PD‐1 pathway blockade can enhance HIV‐specific immunoglobulin production, which restores Tfh help to B cells during HIV infection.^344^ In humanized mice, blocking the PD‐1 pathway induces a progressive reduction in HIV viral load and a significant increase in the percentage of CD4^+^ T cells compared with untreated mice.^416^ Furthermore, PD‐1 blockade enhances HIV‐1‐specific T cell function.^416^

### 5.4 Other therapies

In addition to the aforementioned therapies, various other therapies based on CD4^+^ T cells have been developed (Figure 5D). In patients with prostate cancer in situ, immune checkpoint inhibitors increase the number of tumor‐infiltrating Th1 cells; however, in patients with bone metastasis of prostate tumor, immune checkpoint inhibitors increase Th17 subsets but not Th1 due to the presence of TGF‐β.^368^ Therefore, the combination of anti‐CTLA‐4 and anti‐TGF‐β treatment allows Th1 development and augments the efficacy of ICB.^368^ Meanwhile, it has also been reported that blocking TGF‐β signaling, such as TGF‐β receptor II (TGF‐βRII), in CD4^+^ T cells mediates tumor regression by promoting the remodeling of the blood vasculature, which means a mature and organized tumor vasculature.^417^, ^418^ Notably, CD27 agonism has been reported to mimic CD4^+^ T cell help acting on CD8^+^ T cells to enhance CTL responses.^164^ Moreover, CD27 antibody has been proven to be safe in clinical trials.^370^ Interestingly, the combination of CD27 agonism and PD‐1 blockade recapitulates CD4^+^ T cell help and improves the efficacy of the immunotherapy, suggesting that CTLs primed by CD27 agonism may also experience immune suppression induced by the PD‐1/PD‐L1 pathway in tumors, which can be reversed by PD‐1 blockade.^164^ However, circulating PD‐1^+^CD8^+^ T cells express CD27 after PD‐1 blockade, indicating that CD27 agonism may synergize with PD‐1 blockade via a different mechanism.^371^, ^372^, ^419^

In metastatic breast cancer, CD25‐blocking monoclonal antibody daclizumab can mediate reduction and dysfunction of CD25^hi^CD45RA^neg^ Treg cells and reinvigorate the capability to secrete IFN‐γ. The combination of daclizumab and cancer vaccine can induce a robust CD8^+^ and CD4^+^ T cell priming and boosting and a decline of Treg cells.^420^ The p110δ inactivation in Tregs cells can mediate elimination of tumor cells and reinvigorate CD8^+^ cytotoxic T cells in multiple murine tumor models.^421^ CCR4^+^CD45RA^−^FOXP3^hi^CD4^+^ Treg cells (effector Treg cells, eTreg cells) are predominately among tumor‐infiltrating Treg cells in melanoma. The treatment of anti‐CCR4 mAb mediates a reduction of eTreg cells and upregulation of antigen‐specific CD8^+^ T cells response.^422^

During HIV infection, IL‐10 is upregulated and impairs CD4^+^ T cells activation.^327^, ^328^ Blockade of the IL‐10/IL‐10 receptor pathway mediates viral clearance and functional improvement in T cells.^327^, ^328^ Furthermore, IL‐10 blockade can also significantly enhance the proliferation of HIV‐specific CD4^+^ T cells and elevate the secretion of IFN‐γ and IL‐2.^327^ PD‐L1 blockade restores IFN‐γ, IL‐2, and IL‐13 production, while blockade of IL‐10 receptor pathway remarkably enhances IFN‐γ secretion.^423^ The combination of PD‐L1 blockade and IL‐10Rα blockade mediates a dramatic increase in IFN‐γ and an upregulation of IL‐2, and IL‐13 production in HIV‐specific CD4 T cells.^423^

## 6 CONCLUSION

CD8^+^ T cells have received considerable attention in tumor immunotherapy strategies due to the important role of CTL cells in the killing effect of tumor cells and chronic viruses. In TME and chronic viral infections, the function of CD8^+^ T cells gradually decreases, and the expression of inhibitory receptors, such as the immune checkpoint of PD‐1 and CTLA‐4, gradually increases. In advanced stages of exhaustion, CD8^+^ T cells may even disappear. Additionally, many CD8^+^ T cell‐based immunotherapies, such as ICB, cannot completely reverse the progression of exhaustion. Moreover, when MHC‐I epitope‐based vaccines are utilized, persistent tumor antigens accelerate the exhaustion of CD8^+^ T cells.

CD4^+^ T cells also play an important role in antitumor immunity and antichronic viral immunity owing to their auxiliary effects. Helping signals from CD4^+^ T cells are delivered by DCs to CD8^+^ T cells. This auxiliary effect acts on CD8^+^ T cells through costimulatory molecules, cytokines, or chemokines to affect the activation, proliferation, differentiation, and migration of CD8^+^ T cells. In addition to this auxiliary effect, some of the CD4^+^ T cells have a direct killing function in the antitumor and antichronic viral responses. Moreover, CD4^+^ T cells interact with B cells and induce affinity‐matured and class‐switched plasma cells to regulate antitumor and antichronic viral humoral immunity. On the contrary, Treg cells mediate immunosuppression in the antitumor and antichronic viral activities.

Thus, immunotherapies have been developed based on the mechanisms of CD4^+^ T cells. Specifically, the adoptive transfer of CD4^+^ T cells or CD4^+^ CAR T cells can induce tumor regression and a reduction in chronic viral infections, and these cells display both cytotoxicity and polyfunctionality. MHC‐II‐restricted epitope‐based vaccines can also mediate tumor eradication in different types of tumors and prolong survival. Additionally, the CTLA‐4 blockade strategy can reverse the immunosuppression effect mediated by Treg cells. Moreover, since DCs play an important role in delivering help signals from CD4^+^ T cells, DC‐based vaccines and the CD27 agonism have recently been exploited to inhibit tumor growth.

## AUTHOR CONTRIBUTION

L. X., J. F., J. Y., W. Z., and Z. H. conceived, edited, and wrote this manuscript. H. W. and L. Y. revised this manuscript. All authors have read and approved the final manuscript.

## CONFLICT OF INTEREST STATEMENT

The authors declare they have no conflicts of interest.

## ETHICS STATEMENT

The author declare that ethics approval was not needed for this study.

## Data Availability

Not applicable.
